# Effects of Blood Collection Conditions on Ovarian Cancer Serum Markers

**DOI:** 10.1371/journal.pone.0001281

**Published:** 2007-12-05

**Authors:** Jason D. Thorpe, Xiaobo Duan, Robin Forrest, Kimberly Lowe, Lauren Brown, Elliot Segal, Brad Nelson, Garnet L. Anderson, Martin McIntosh, Nicole Urban

**Affiliations:** 1 Fred Hutchinson Cancer Research Center, Seattle, Washington, United States of America; 2 Onco Detectors International LLC, Bethesda, Maryland, United States of America; 3 BC Cancer Agency, Trev and Joyce Deeley Research Centre, Victoria, British Columbia, Canada; 4 Public Health Sciences Division, Fred Hutchinson Cancer Research Center, Seattle, Washington, United States of America; Menzies School of Health Research, Australia

## Abstract

**Background:**

Evaluating diagnostic and early detection biomarkers requires comparing serum protein concentrations among biosamples ascertained from subjects with and without cancer. Efforts are generally made to standardize blood processing and storage conditions for cases and controls, but blood sample collection conditions cannot be completely controlled. For example, blood samples from cases are often obtained from persons aware of their diagnoses, and collected after fasting or in surgery, whereas blood samples from some controls may be obtained in different conditions, such as a clinic visit. By measuring the effects of differences in collection conditions on three different markers, we investigated the potential of these effects to bias validation studies.

**Methodology and Principle Findings:**

We analyzed serum concentrations of three previously studied putative ovarian cancer serum biomarkers–CA 125, Prolactin and MIF–in healthy women, women with ovarian cancer undergoing gynecologic surgery, women undergoing surgery for benign ovary pathology, and women undergoing surgery with pathologically normal ovaries. For women undergoing surgery, a blood sample was collected either in the clinic 1 to 39 days prior to surgery, or on the day of surgery after anesthesia was administered but prior to the surgical procedure, or both. We found that one marker, prolactin, was dramatically affected by collection conditions, while CA 125 and MIF were unaffected. Prolactin levels were not different between case and control groups after accounting for the conditions of sample collection, suggesting that sample ascertainment could explain some or all of the previously reported results about its potential as a biomarker for ovarian cancer.

**Conclusions:**

Biomarker validation studies should use standardized collection conditions, use multiple control groups, and/or collect samples from cases prior to influence of diagnosis whenever feasible to detect and correct for potential biases associated with sample collection.

## Introduction

We hypothesize that even with identical sample processing and storage protocols the environment and conditions of sample collection can affect the levels of biomarkers, and that these potential biases should be anticipated in biomarker validation study design. Specifically, the environment surrounding diagnosis and collection of specimens from cases, such as surgical preparation, may affect blood chemistry in a way that introduces systematic changes that may be mistakenly attributed to the disease state. We demonstrate these effects by evaluating conditions of blood collection in one established and two novel ovarian cancer serum markers: CA 125, Prolactin, and Macrophage Migration Inhibitory Factor (MIF). We show that CA 125 and MIF behave as previously reported but that prolactin's performance is strongly affected by biases in sample ascertainment.

Cancer early detection biomarker validation studies are designed to determine which proteins can distinguish between healthy people and those with cancer. In contrast, a diagnostic marker intends to distinguish between people with cancer and those with benign conditions. To potentially impact cancer mortality a marker must show abnormal levels in the blood of cases compared to their appropriate controls, and for early detection purposes they must elevate early enough in the disease process to identify the disease at an early and more treatable state [1]. Evaluating a protein in pre-clinical specimens collected well before suspicion or diagnosis of cancer would be ideal for early detection studies, whereas samples obtained at clinical presentation of disease are most relevant for diagnostic markers. However, because pre-clinical specimens are seldom available, especially for rare diseases, first-phase early detection validation studies often seek to determine whether or not a marker can distinguish persons with symptomatic disease from healthy controls prior to further investment [2].

The primary intent of our biomarker validation study is to ascertain to what extent the classification performance of a biomarker can be attributed to disease associated response rather than to ascertainment biases in sample collection. It is common to construct case and control groups that are matched on sample collection protocols, storage duration, subject age, and other epidemiological information, in order to reduce potential biases related to these factors. Less emphasis has been placed on using multiple sources of control or case groups in order to detect potential biases or on using procedures that may adjust for biases, such as conditions of sample collection. In this manuscript we demonstrate the potential value in conducting biomarker validation studies using multiple sources of well annotated case and control groups. We demonstrate that prolactin is highly sensitive to the conditions of collection: after adjusting for the conditions of collection the marker is no longer considered a viable candidate. CA 125 and MIF are shown to not be highly susceptible to these conditions.

We selected three markers–CA 125, Prolactin and MIF–to evaluate in a highly annotated set of case and control specimens.

CA 125 is a mucin-like glycoprotein which has been shown to be elevated in most women with OC compared to a healthy population [3]. CA 125 has also been evaluated in preclinical serum specimens, and each study suggests that CA 125 is a predictive marker that becomes increasingly powerful with proximity to diagnosis [4]–[6]. However, CA 125 is also elevated in several benign conditions and may also be a marker of inflammation [7]. Due to insufficient sensitivity and specificity, CA125 is not used clinically as a stand alone screening test. Falling CA 125 levels after treatment are used to confirm response to specific treatments [8] and elevating CA 125 levels signal recurrence [9]. CA 125 is a ligand of Mesothelin [10], which may play a role in the metastasis of OC to the peritoneum [11].

MIF is a proinflammatory cytokine which has been identified as a candidate early detection marker for OC [12], although analysis of its performance as a biomarker for early stage ovarian cancer suggested that it does not exhibit higher sensitivity or specificity than CA 125 [13]. Inhibition of the anti-inflammatory properties of glucocorticoids is an important effect of MIF [14], [15]. MIF may also mediate some of the stimulatory effects of inflammation on cancer progression. Evidence of MIF's role in the regulation of tumor-suppressor genes such as p53 [16], [17] and angiogenesis [18], [19] points to a potential link between chronic inflammation and the development of cancer.

Prolactin has been identified as a candidate early detection marker for ovarian cancer with reports of impressively high sensitivity (>90%) and specificity (>98%) [12]. Elevated levels of circulating prolactin (hyperprolactinemia) have long been associated with pituitary tumors [20], but more recently prolactin has been reported in association with a variety of additional cancers, including breast [21]–[23], prostate [24], and colon carcinoma [25].

## Methods

### Study population and serum specimen collection

Serum samples were collected by the Pacific Ovarian Cancer Research Consortium for use in biomarker validation experiments. The samples used in this study were collected at Swedish Medical Center or Virginia Mason Hospital (Seattle, WA, USA) between July 1, 2004 and June 30, 2006. Participants were recruited from the following populations: apparently healthy women attending regular breast cancer screening exams (healthy controls), women undergoing gynecologic surgery for a variety of conditions but with normal ovarian pathology (surgical controls), women without malignancy but with benign ovarian disease (benign controls), and women diagnosed with ovarian cancer, fallopian tube cancer, or primary peritoneal invasive cancer. Identical specimen processing protocols were used for all groups.

A sample of subjects from each of these conditions was selected for biomarker validation studies. Patients with prior oophorectomy or diagnosis of ovarian cancer were excluded from the study population. Cases included 50 consecutively recruited patients with ovarian (n = 45), fallopian tube (n = 1), and peritoneal cancer (n = 4). Control groups included healthy controls (n = 36), surgical controls (n = 14), and benign controls (n = 30). The validation study was powered to detect a marker with 30% sensitivity at 95% specificity, or better. Demographics of the patients included in this study are described in **Supplementary Tables S1** and **S2.**

The healthy, surgical and benign controls used in this study were selected from larger control populations (n = 346, 63, and 38 respectively) to match the cases on age, race, family history of ovarian and breast cancer [26], and blood collection date. We used propensity score matching to balance the overall distribution of the groups [27]. Briefly, a propensity score was estimated by predicting case status using logistic regression on each of the variables of interest. After first selecting the case group, individual controls were selected that most closely matched a randomly identified member of the case group on the assigned propensity score until pre-specified numbers for each control group had been selected.

Participants in the surgical control, benign control and case populations donated serum specimens either at a pre-surgical appointment 1 to 39 days prior to surgery or on the day of surgery after administration of anesthesia but before surgical treatment or chemotherapy. To maximize the power to detect differences in marker levels due to conditions of collection, we included specimens collected both on the day of surgery and at the pre-surgical appointment from the same patient (n = 30) whenever possible. Participants in the healthy control population donated blood at a regular mammography screening appointment.

### Laboratory methods

#### Prolactin and MIF Assays

Serum levels of prolactin and MIF were measured by ELISA using kits acquired from Diagnostic Systems Laboratories (Webster, TX) and Onco Detectors International LLC (Bethesda, MD) respectively. Assays were performed according to manufacturer's instructions. The concentrations of human prolactin and MIF were determined using a linear standard curve that was constructed by plotting the mean absorbance against the known concentration for each reference standard. See Text S1 for details.

#### CA 125 Assay

Serum levels of CA 125 were measured by bead-based immunoassays as previously described [28] using anti-CA 125 mouse monocolonal antibodies ×306 (capture) and ×52 (detection) acquired from Research Diagnostics, Inc (RDI, Flanders, NJ). Readings from the immunoassay were normalized and then z-scores were calculated by centering and scaling observations so that healthy controls have mean 0 and variance 1. See Text S1 for details.

Specimens were randomized onto two plates with 80 specimens each, and laboratory personnel were blinded to case status at all times.

### Statistical analysis

Receiver operating curves (ROC) were used to determine if serum marker concentrations discriminated between cases and healthy controls [29]. The area under each ROC curve (AUC) was calculated and significance for marker discrimination (AUC different from 0.5) was determined using the Mann-Whittney U statistic. ROC curves for healthy control samples and case samples collected either prior to surgery or on the day of surgery for each marker were compared using the method described by Metz et al [30].

To evaluate whether marker levels differed between case and control groups after adjusting for conditions of blood collection, we fitted multiple linear regression models to each marker as the dependent variable with indicator variables for each case/control population and an indicator variable for conditions of blood sample collection (clinic visit or in surgery) as independent variables. The regression model for the *i* ^th^ woman at time *t* was:
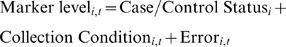



The reference group in each model is the healthy control group. This model can potentially separate the components of variance due to conditions of sample collection and presence of malignancy. In particular, for markers that elevate due to the presence of ovarian cancer and are also affected by the conditions of blood collection, each effect can be estimated from the model parameters. Regressions were performed using Generalized Estimating Equations (GEE) methods to avoid bias in estimates of standard errors because marker levels were measured twice for 30 women in the study.

P-values for differences between partially correlated ROC curves were calculated with the ROCKIT software package[31] using the bivariate test. All other calculations were performed using the R statistical programming language[32].

## Results

**Figure 1:**
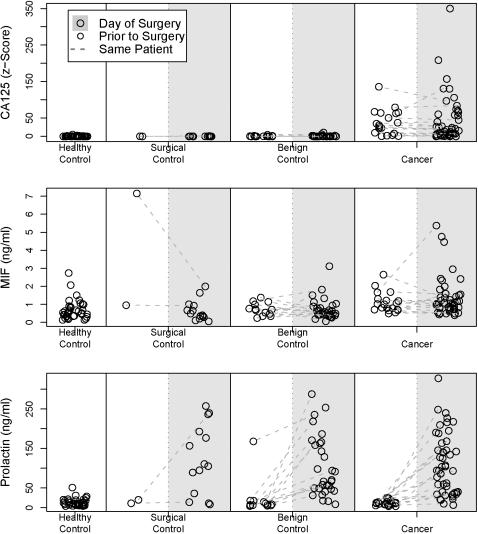
Prolactin, MIF and CA 125 levels stratified by population and surgical status. Dotted lines connect surgical and pre-surgical marker levels measured within the same women under both surgical and non-surgical conditions

**Figure 2:**
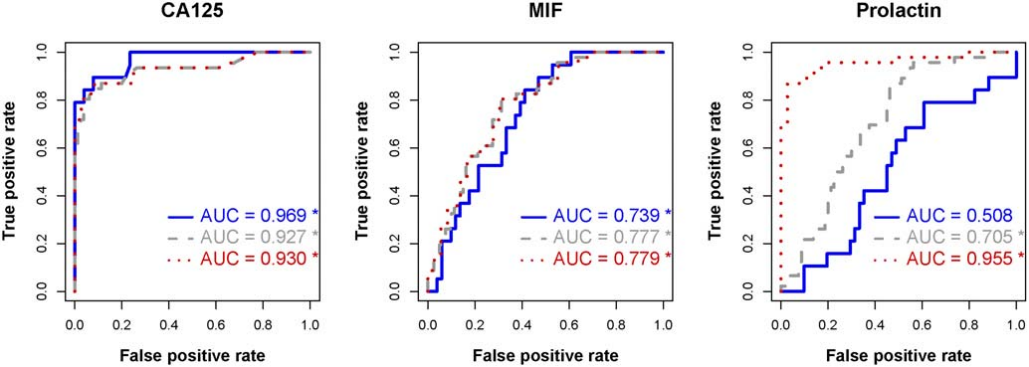
ROC curves comparing marker concentrations in cases to healthy controls. Case specimens were obtained either at surgery (surgical comparison; dashed line) or 1 to 39 days prior to surgery (pre-surgical comparison; solid line). The pre-surgical comparison suggests that prolactin levels do not discriminate between women with and without cancer in the clinic setting. * indicates AUC different from 0.5 at alpha = 0.05 significance level (Mann Whitney U test)

Marker levels from each case/control group collected in surgery and at the pre-surgical clinic visit are shown in **Figure 1** and summarized in **Table 1**. ROC analysis showed that CA 125 and MIF concentrations discriminate between healthy controls and cases collected either at surgery or 1 to 39 days prior to surgery (figure 2a ,b; p<0.05 for each marker and condition). Moreover, the AUCs were not significantly different between the two collection conditions (figure 2a,b; p = 0.297 and 0.416 respectively).

**Table 1:** Summary of marker levels by case/control group and collection conditions.

		Collection Conditions			
		1 to 39 Days Before Surgery		At Surgery	
Marker	Case/Control Group	n	median (5^th^, 95^th^ percentile)	n	Median (5^th^, 95^th^ percentile)
CA 125 (z-Score)	Healthy Control	36	−0.335 (−0.747, 4.702 )	–	–
	Surgical Control	2	−0.396 (−0.612, −0.18 )	14	−0.369 (−0.747, 0.262 )
	Benign Control	13	−0.276 (−0.814, 3.774 )	30	−0.327 (−0.814, 10.084 )
	Cancer	19	30.198 (0.112, 135.667 )	46	16.151 (−0.473, 350.168 )
MIF (ng/mL)	Healthy Control	36	0.5 (0.2, 1.6 )	–	–
	Surgical Control	2	4.1 (1.3, 6.8 )	14	0.4 (0.1, 1.8 )
	Benign Control	13	0.7 (0.3, 1.3 )	30	0.6 (0.2, 1.7 )
	Cancer	19	1 (0.5, 2.1 )	46	1 (0.5, 4.1 )
Prolactin (ng/mL)	Healthy Control	36	9.9 (4.9, 29.3 )	–	–
	Surgical Control	2	15.8 (11.8, 19.7 )	14	108.1 (10.4, 246.2 )
	Benign Control	13	7.7 (5, 78.1 )	30	68.6 (17.3, 245.6 )
	Cancer	19	10.8 (3.9, 24.3 )	46	99.2 (20, 236.8 )

Serum specimens were collected from healthy controls at a regular mammography screening appointment. Specimens were collected from the remaining populations either at a pre-surgical appointment 1 to 39 days prior to surgery or on the day of surgery after administration of anesthesia but before the surgical procedure.

Prolactin levels were highly elevated in the case specimens collected at surgery (figure 1c) and prolactin levels discriminated between case specimens collected at surgery and healthy controls with high sensitivity and specificity (figure 2c, dotted line). However, this difference disappeared when we compared case specimens collected 1 to 39 days prior to surgery to the healthy controls (figure 2c, solid line). The AUC for discriminating between cases and controls was significantly lower in specimens collected in the short interval prior to surgery than for the specimens obtained at surgery (figure 2c, p_difference in AUC_<0.0005). Moreover, serum prolactin levels did not discriminate between healthy controls and case specimens collected 1 to 39 days prior to surgery (figure 2c, solid line AUC = 0.497).

We used multiple linear regression models to examine whether differences in marker levels were associated with case status and/or with conditions of blood sample collection. In the regression models, CA 125 and MIF concentrations were not significantly affected by the conditions of blood collection (table 2, p = 0.60 and 0.71 respectively) and were elevated in the cases relative to the healthy controls (table 2, p<0.005 for each marker). Prolactin levels, however, were significantly increased in serum samples collected at surgery (table 2, p<0.005) and after adjusting for conditions of blood collection, prolactin was not elevated in cases relative to healthy controls (table 2, p = 0.69). These data suggest that the differences observed with prolactin can be attributed entirely to blood collection conditions, with no residual signal associated with malignancy.

**Table 2:** Results from the multiple linear regression models

Marker	Variable	Level	Estimate	Std Err	p-Value
**CA125**	Blood Collection Conditions	At Clinic	(Reference)		
		In Surgery	3.18	6.05	0.6
	Case/Control Group	Healthy Control	(Reference)		
		Surgical Control	−3.11	5.31	0.58
		Benign Control	−1.87	4.24	0.66
		Ovarian Cancer	39.43	7.14	<0.005
**MIF**	Blood Collection Conditions	At Clinic	(Reference)		
		In Surgery	−0.09	0.25	0.71
	Case/Control Group	Healthy Control	(Reference)		
		Surgical Control	0.46	0.65	0.48
		Benign Control	0.11	0.2	0.59
		Ovarian Cancer	0.67	0.22	<0.005
**Prolactin**	Blood Collection Conditions	At Clinic	(Reference)		
		In Surgery	93.23	8.82	<0.005
	Case/Control Group	Healthy Control	(Reference)		
		Surgical Control	15.23	20.28	0.45
		Benign Control	0.45	11.49	0.97
		Ovarian Cancer	2.37	5.97	0.69

Multiple linear regression models were fitted to each marker as the dependent variable with indicator variables for each case/control population and an indicator variable for conditions of blood sample collection (clinic visit or in surgery) as independent variables. GEE methods were used to avoid bias in estimates of standard errors because marker levels were measured twice for 30 women in the study

## Discussion

The approach of using commercially available assays to validate candidate biomarkers is very promising. However, results can be misleading if conditions of the blood sample collection for cases and controls are not standardized or otherwise accounted for. We show here that serum prolactin levels are strongly influenced by the conditions of blood collection and that prolactin does not discriminate between cancer and non-cancer patients in serum specimens collected similarly in a clinic setting. In contrast, CA 125 and MIF were not affected by the conditions of blood collection; both markers discriminated between cases and controls irrespective of whether serum specimens were collected at surgery or in a short interval prior to surgery.

This finding is consistent with previous reports that prolactin levels elevate during surgery and post-operatively in female patients undergoing surgery with halothane (general) anesthesia [33]. Prolactin levels are also elevated in rats undergoing general anesthesia with pentobarbital, regardless of surgery [34]. In our study, specimens collected on the day of surgery were obtained after general anesthesia was administered but before any incisions were made. Serum prolactin levels at surgery may have been affected by anesthesia or by other conditions of surgery such as stress [20].

In multiple linear regression models, differences in CA125 and MIF levels were associated with case status but not by the conditions of sample ascertainment. For prolactin, the reverse was true suggesting that prolactin levels are affected by the conditions of surgery and may not be a marker of ovarian cancer. These multivariate analyses complemented the ROC analyses by adjusting for the conditions of blood collection, thus allowing for the possibility that a marker signals malignancy despite being affected by the conditions of blood collection. Adjustment for collection conditions in the analysis is useful more generally when blood samples collected under identical conditions are not available from every participant in a study.

The use of multiple sources of control specimens collected under various conditions may alert researchers to potential biases. We have demonstrated that permitting collection conditions to vary in cases and controls but using correct annotations may alert researchers to potential problems. Whenever it is not feasible to obtain multiple collections from cases (both within and outside of surgery) the use of surgical controls can be used as a screen for the possible effects of collection condition. For example, it can be seen in figure 1c that prolactin levels are higher in the control groups where samples were collected at surgery than in healthy controls, again suggesting that elevated prolactin levels may not be specific to malignancy.

The limited availability of pre-clinical specimens from ovarian cancer patients presents a significant challenge to researchers trying to discover or validate novel biomarkers for early detection. The majority of specimens from cancer patients that are available for research are not collected from women or clinicians who are blind to their impending diagnosis. Our results illustrate that biases between case and control populations can lead to false positive experimental results and that controlling for conditions of blood collection can reduce false discovery and false validation in biomarker experiments. It is important to detect, and whenever possible to correct for, biases in conditions of blood collection when attempting to discover and validate novel biomarkers.

## Supporting Information

Table S1 Summary of patient demographics by case status
(0.03 MB DOC)

Table S2 Summary of ovarian cancers by stage and histology
(0.03 MB DOC)

Text S1 Detailed Descriptions of Assay Procedures
(0.03 MB DOC)
