# Supplementary material for: Effects of Blood Collection Conditions on Ovarian Cancer Serum Markers
Source: PLoS One. 2007 Dec 5;2(12):e1281. doi: 10.1371/journal.pone.0001281 (PMC2093996; doi:10.1371/journal.pone.0001281)
Supplement: Table S1 — Summary of patient demographics by case status (0.03 MB DOC) [file pone.0001281.s001.doc]

**Table S1. Summary of patient demographics by case status**

| Variable | Subcategory | Healthy | Surgical Controls | Benign Controls | Cases |
| --- | --- | --- | --- | --- | --- |
| N |  | 36 | 14 | 30 | 50 |
| Age (sd) |  | 57.8 (10.0) | 59.6 (13.2) | 53.3 (11.8) | 58 (12.9) |
| Age > 50 |  | 25 (69.4%) | 8 (57.1%) | 22 (73.3%) | 35 (70.0%) |
| Significant family history* or BRCA1 or BRCA2 mutation |  | 5 (13.8%) | 1 (6.6%) | 0 (0%) | 9 (18.0%) |
| Specimens Available | Pre-Surgical Only | 36 (100%) | 0 (0%) | 0 (0%) | 4 (8.0%) |
|  | Surgical Only | -- | 12 (85.7%) | 17 (56.7%) | 31 (62%) |
|  | Both | -- | 2 (14.3%) | 13 (46.3%) | 15 (30%) |

Surgical specimens were collected after administration of anesthesia but prior to the surgical procedure. Pre-surgical specimens were collected at a pre-surgical appointment 1 to 39 days prior to surgery.

*Significant family history of ovarian or breast cancer was defined as (*a*) two or more breast cancer cases and one or more ovarian cancer cases diagnosed at any age, among first- or second-degree relatives of the same lineage; (*b*) three or more breast cancer cases diagnosed before the age of 50, among first- or second-degree relatives of the same lineage; (*c*) a family history that includes sister pairs with two of the following cancers: two breast cancers diagnosed before the age of 50 or a breast cancer diagnosed before the age of 50 and an ovarian cancer diagnosed at any age; or (*d*) two or more ovarian cancer cases (any age) among first- or second-degree relatives of the same lineage
