# Supplementary material for: Effects of Blood Collection Conditions on Ovarian Cancer Serum Markers
Source: PLoS One. 2007 Dec 5;2(12):e1281. doi: 10.1371/journal.pone.0001281 (PMC2093996; doi:10.1371/journal.pone.0001281)
Supplement: Table S2 — Summary of ovarian cancers by stage and histology (0.03 MB DOC) [file pone.0001281.s002.doc]

**Table S2. Summary of ovarian cancers by stage and histology**

|  | FIGO Stage | | | | | |
| --- | --- | --- | --- | --- | --- | --- |
| Histology | Stage 1 | Stage 2 | Stage 3 | Stage 4 | Unstaged | All Stages |
| Clear Cell | 2 | 1 | 3 |  |  | 6 |
| Endometrioid | 3 | 2 |  |  |  | 5 |
| Mucinous | 3 |  |  |  |  | 3 |
| Other | 2 |  |  |  | 1 | 3 |
| Serous | 1 | 2 | 23 | 7 |  | 33 |
| All histologies | 11 | 5 | 26 | 7 | 1 | 50 |
