# Supplementary material for: Effects of Blood Collection Conditions on Ovarian Cancer Serum Markers
Source: PLoS One. 2007 Dec 5;2(12):e1281. doi: 10.1371/journal.pone.0001281 (PMC2093996; doi:10.1371/journal.pone.0001281)
Supplement: Text S1 — Detailed Descriptions of Assay Procedures (0.03 MB DOC) [file pone.0001281.s003.doc]

### Detailed Descriptions of Assay Procedures

### Prolactin and MIF Assays

Serum levels of prolactin were measured by ELISA using kits acquired from Diagnostic Systems Laboratories (Webster, TX). The prolactin ELISA reportedly has a linear detection range of 2.1 – 158.0 ng/ml. Assays were performed following manufacturers’ instructions. Briefly, 25μl of serum, standard or control was added to each well followed by 100μl of assay buffer. Specimens were then incubated at room temperature with shaking for one hour. After washing, 100μl of antibody/HRP conjugate solution was added and plates were incubated for one hour. After further washing, 100 μl TMB substrate solution was added to each well. After incubating for 10 minutes, the reactions were stopped by adding 100 µL 0.2 N sulfuric acid.

Serum levels of MIF were measured by ELISA using kits acquired from Onco Detectors International LLC (Bethesda, MD). The MIF ELISA reportedly has a lower detection limit of 0.1ng/ml. Assays were performed following manufacturers’ instructions. Briefly, serum was diluted 1:10 in sample diluent, and 100μl of diluted serum was added to each well. After incubating for 2 hours at room temperature, plates were washed and incubated with biontinylated secondary antibody for 2 hours. After further washing, 100μl of diluted streptavidin-HRP was added to each well, followed by a 20 minute incubation. After a third round of washing, 100μl TMB substrate solution was added to each well and the plates were incubated for 20 min. The reaction was stopped by adding 100 μl 1N HCL to each well.

Color intensity was determined using a SpectraMax M2 Microplate Reader (Molecular Devices, Sunnyvale, CA) at 450nm with the appropriate baseline correction for each assay.

### CA 125 Assay

Serum levels of CA 125 were measured by bead-based immunoassay using anti-CA 125 mouse monocolonal antibodies (mAbs) X306 (capture) and X52 (detection) acquired from Research Diagnostics, Inc.(Flanders, NJ). The mAbs were dialyzed against Dulbecco’s phosphate buffered saline (PBS) (Invitrogen Corporation, Carlsbad, CA) when necessary. Anti-CA 125 X52 was biotinylated using the EZ-Link-sulfo-NHS-biotinylation kit (Pierce, Rockford, IL) according to the manufacturer’s instructions and dialyzed (G Biosciences Tube-O-Dialyzer, 4kDa MWCO) against PBS.

Carboxy-coated microspheres were coupled with 5 µg/ml capture mAb, using the Bio-Plex Amine Coupling Kit (Bio-Rad Laboratories, Inc., Hercules, CA). Bead couplings and bead-based immunoassays were performed as previously described [32]. Briefly, assays were performed using the Bio-Plex Cytokine Assay Kit (Bio-Rad) in 96-wellfilter plates (Millipore Corporation, Billerica, MA) with a vacuum manifold (Millipore) to drain reagents and for wash steps. All incubations were performed at room temperature in the dark and on a plate shaker. Serum was diluted 4-fold in Bio-Plex Human Serum Diluent (Bio-Rad) and detected with 2 µg/ml biotinylated X52 followed by 100-fold diluted phycoerythrin-conjugated streptavidin (Bio-Rad). The median fluorescence intensity (MFI) of 100 microspheres from each sample was analyzed with the Bio-Plex Array reader (Bio-Rad).

To reduce plate-to-plate variation in the assay, readings were normalized using negative and positive control sera formed by pooling serum from seven healthy women and women diagnosed with ovarian cancer, respectively. One part positive control pool was diluted with 3 parts negative control pool to form an intermediate pool. Five replicates of the intermediate pool were included on each plate. Readings from patient samples were normalized by dividing by the average MFI from the intermediate pool replicates included on the same plate. Separate experiments showed that this procedure reduces plate-to-plate variation in the results (data not shown). After normalization, z scores were calculated by centering and scaling observations so that healthy controls have mean 0 and variance 1.
